# Exceedingly Rare Bilateral Synchronous Germ Cell Testicular Tumors With Different Histopathological Features

**DOI:** 10.7759/cureus.42374

**Published:** 2023-07-24

**Authors:** Jong H Kim, Ray D Page, Gregory A Moses, Rod C Columbres

**Affiliations:** 1 Internal Medicine, Medical City Weatherford, Weatherford, USA; 2 Oncology, Center for Cancer and Blood Disorders, Fort Worth, USA; 3 Pathology and Laboratory Medicine, Medical City Weatherford, Weatherford, USA; 4 Urology, William Carey University College of Osteopathic Medicine, Hattiesburg, USA

**Keywords:** testicular seminoma, primary choriocarcinoma, yolk sac tumor, embryonal cell carcinoma, mixed germ cell tumor, bilateral synchronous testicular tumor, bilateral testicular tumors, bilateral testicular cancer

## Abstract

Bilateral synchronous testicular tumors are a relatively uncommon occurrence, especially when they involve germ cell tumors of different histology. In this context, we present a compelling case report of a male patient who was diagnosed with bilateral synchronous germ cell testicular tumors, with one being a seminoma and the other a non-seminomatous germ cell tumor (NSGCT). The coexistence of two distinct histological types, seminoma and NSGCT, necessitates a comprehensive diagnostic approach to accurately identify and characterize each tumor. This underscores the importance of clinical history, physical examination, imaging techniques, and histopathological analysis to establish an appropriate diagnosis. Careful consideration must be given to factors such as tumor stage, histological subtype, and individual patient characteristics to determine the most suitable treatment strategy. Treatment options may encompass a combination of surgery, chemotherapy, and radiation therapy, tailored to each tumor's specific characteristics and the patient's overall health. By highlighting this unique case, we aim to underscore the significance of meticulous evaluation and accurate diagnosis when confronted with bilateral synchronous testicular tumors of different histology.

## Introduction

Testicular cancer poses a significant health risk, especially among young men aged 15 to 35 years. It is considered the most common solid malignancy in this age group, with an estimated annual incidence of six cases per 100,000 men in the United States [1]. While bilateral synchronous testicular tumors are relatively rare, accounting for only about 2-3% of cases, they are predominantly observed as metachronous tumors. However, in the rare instances when bilateral synchronous tumors do occur, they represent approximately 10% of bilateral testicular tumors [2].

These cases often exhibit histological similarities between the tumors, with bilateral seminoma being the most frequently encountered type, as Koppad et al. presented a case that illustrated bilateral synchronous seminomas with the same histopathological origin [3]. It is worth mentioning that the occurrence of discordant histological patterns in bilateral testicular tumors is an extremely rare phenomenon, with fewer than 100 documented cases reported in the existing literature [4]. The understanding and documentation of these exceptional cases contribute to our knowledge and clinical understanding of testicular cancer, leading to improved diagnostic, treatment, and management strategies.

This report presents an additional case of synchronous primary bilateral testicular tumors, each exhibiting distinct histopathology. Given the rarity of synchronous bilateral testicular neoplasms, it is important to emphasize that their treatment approaches are unique and should be tailored based on the tumor stage. These cases should be managed separately with close surveillance in accordance with the guidelines provided by the National Comprehensive Cancer Network (NCCN) [5]. 

This article was previously presented as a poster at the 2023 North Texas GME (Graduate Medical Education) Research Forum on April 28, 2023.

## Case presentation

**Figure 1:**
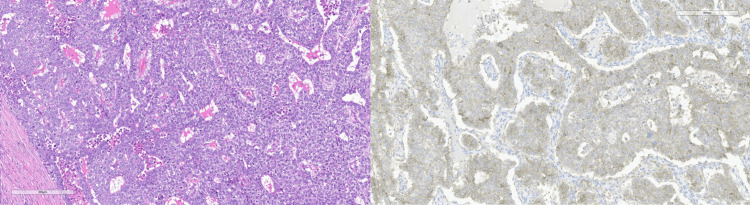
Right-sided embryonal carcinoma with H&E (left) and CD30 immunohistochemical stain (right) H&E: Hematoxylin and Eosin

**Figure 2:**
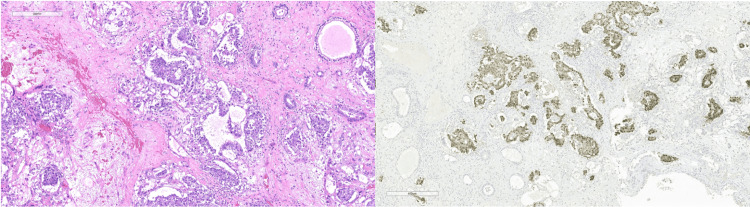
Right-sided yolk sac tumor with H&E (left) and OCT 3/4 immunohistochemical stain (right) H&E: Hematoxylin and Eosin; OCT: Octamer Binding Transcription Factor

**Figure 3:**
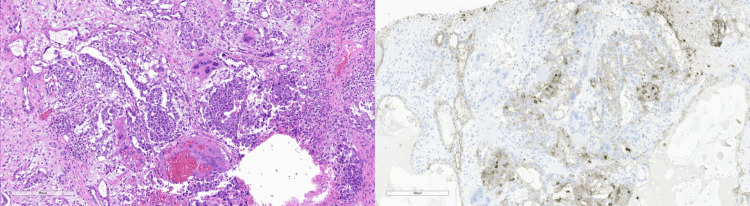
Right-sided choriocarcinoma with H&E (left) and PLAP immunohistochemical stain (right) H&E: Hematoxylin and Eosin; PLAP: Placental Alkaline Phosphatase

**Figure 4:**
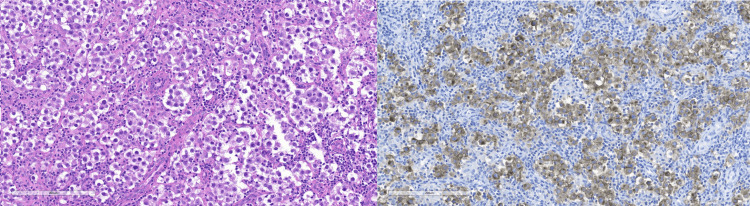
Left-sided seminoma with H&E (left) and CD117 immunohistochemical stain (right) H&E: Hematoxylin and Eosin

A 43‐year‐old man with no significant past medical history presented to his primary care physician with increasing right testicular pain and swelling. A testicular sonogram showed a large right-sided testicular mass and a 1.7 cm left-sided testicular mass, both suspicious of malignancy. Pre‐surgical laboratories showed 0.97 ng/mL prostate-specific antigen (PSA), 1875 ng/mL alpha-fetoprotein (AFP), 2508 mIU/mL human chorionic gonadotropin (hCG), and 448 IU/L lactate dehydrogenase (LDH). The patient had a right inguinal orchiectomy which revealed a mixed germ cell tumor as depicted in Figures 1, 2, 3 (60% embryonal carcinoma, 30% yolk sac tumor, 10% choriocarcinoma) with clear margins, no invasion, stage IS (pT1b N0 M0). Subsequent left inguinal orchiectomy was performed, which demonstrated complete seminoma as shown in Figure 4, without any invasion with clear margins, stage IA (pT1a N0 M0). Post-surgical staging workup, including computed tomography (CT) scans and positron emission tomography (PET) imaging, showed no evidence of metastasis or lymph node involvement. Based on the stage and histology of the tumors, the patient was recommended to have a scheduled six-month follow-up to monitor for tumor markers and radiographic images.

Following the initiation of bilateral orchiectomy, the patient underwent established protocols for monitoring tumor markers, including AFP and HCG, which normalized to assess surgical response. Additionally, the patient underwent regular imaging evaluations, including CT scans and tumor marker assessments at predetermined intervals to monitor for any evidence of residual disease or to detect any signs of recurrence. Psychosocial support and fertility preservation counseling were provided to the patient due to the impact of the bilateral orchiectomy on his reproductive health. Options such as sperm banking and discussions regarding the potential impact of treatment on future fertility and quality of life were addressed.

## Discussion

We presented a case of a 43‐year‐old man who underwent sequential bilateral orchiectomies for bilateral synchronous testicular tumors right-sided mixed germ cell tumor and left-sided seminoma. It is now widely recognized that bilateral synchronous tumors of different histology typically arise from the development of two separate primary tumors, rather than being a result of metastasis from one testis to the other [6,7]. This updated understanding challenges the previous assumption that bilateral synchronous tumors with different histologies originated from metastatic spread within the testicular tissue. Instead, emerging evidence suggests a multifocal origin, indicating that each tumor develops independently within its respective testis [8,9].

The distinction in the origin of bilateral synchronous tumors, arising independently within each testis, holds significant implications for the clinical management and treatment strategies in testicular cancer. It highlights the importance of evaluating each tumor individually, taking into account its histological characteristics, staging, and potential for metastasis. Accurate histopathological examination and immunohistochemical analysis play a vital role in determining the specific components of each tumor, enabling appropriate treatment selection and prognostic assessment [10,11]. The patient in the present report showed left-sided pure seminoma, and right-sided mixed germ cell tumor with 60% embryonal carcinoma, 30% yolk sac tumor, and 10% choriocarcinoma.

Despite the infrequency of this condition, the treatment approach followed is the standard management of unilateral testicular carcinoma, with the additional consideration of prioritizing the more aggressive tumor component [12,13]. Salazar-Mejia et al. reported that their patient’s poor compliance with surveillance was identified as a risk factor for relapse and poor outcome; the patient underwent one cycle of bleomycin, etoposide, and cisplatin (BEP) for adjuvant chemotherapy. After four years of follow-up, their patient showed no evidence of relapse [13]. Collaboration among urologists, pathologists, radiologists, oncologists, and support services is essential for optimal patient outcomes in the management of testicular tumors. This multidisciplinary approach allows for comprehensive assessment and treatment planning. Long-term follow-up is crucial to monitor for potential recurrence or the development of metachronous tumors [10].

## Conclusions

In this case report, we emphasized the rarity and diagnostic complexities associated with bilateral synchronous germ cell testicular tumors of different histologic findings. The patient was diagnosed with synchronous bilateral testicular tumors with mixed germ cell tumor and seminoma, who underwent sequential orchiectomies and was monitored with tumor markers and radiographic images. Managing such cases requires a comprehensive evaluation, precise histopathological assessment, and adherence to established treatment guidelines for each tumor component. As our understanding of testicular tumors continues to evolve, ongoing research and the accumulation of evidence are vital in refining diagnostic and therapeutic strategies for these complex cases. Sharing case reports, such as the one presented here, contributes to the growing body of knowledge and promotes improved management of rare and challenging presentations in the field of testicular oncology.
